# Data on trend changes of drinking groundwater resources quality: A case study in Abhar

**DOI:** 10.1016/j.dib.2018.01.032

**Published:** 2018-01-31

**Authors:** Mahmood Yousefi, Mohammad Hadi Dehghani, Saba Maghsoudi Nasab, Vahid Taghavimanesh, Shahrokh Nazmara, Ali Akbar Mohammadi

**Affiliations:** a Department of Environmental Health Engineering, School of Public Health, Tehran University of Medical Sciences, Tehran, Iran; b Department of Epidemiology and Biostatistics, School of Public Health, Tehran University of Medical Sciences, Tehran, Iran; c Students Research Committee, Neyshabur University of Medical Sciences, Neyshabur, Iran; d Department of Environmental Health Engineering, Neyshabur University of Medical Sciences, Neyshabur, Iran

**Keywords:** Trend changes, Drinking water, Groundwater, Abhar, Water resources

## Abstract

The data of this study was to determine the groundwater quality trend changes in Abhar city (Iran) during one decade (2002–2016). In the first and end year of the study period, the Mean±SD of total hardness (as calcium carbonate, mg/l), electrical conductivity (as micromhos/cm) and total dissolved solid in the first and end year of the study period were 192.69±56.83, 235.25±84.73 and 606.21±194.69, 744.55±288.52 and 348.79±106.81, 464.71±183.52 respectively. On the basis of Pearson correlation coefficient, the ascending trend of some parameters concentration with time was significant at the level of 95% of confidence limits (α ≤ 0.05).

**Specifications Table**

**Table:** 

Subject area	Chemistry
More specific subject area	Describe narrower subject area
Type of data	Table, Figures, Charts
How data was acquired	The required data were collected from the results recorded in the water in the Water and Wastewater Company of Zanjan province during the years 2002–2016
Data format	Raw, Analyzed
Experimental factors	All water samples in polyethylene bottles were stored in a dark place at room temperature until the metals analysis
Data source location	Abhar, Zanjan province, Iran
Data accessibility	Data are included in this article and supplement file excel

**Value of data**

• Determination of the physical and chemical parameter including EC, pH, TDS, TH, Ca^2+^, Mg^2+^, $HCO_{3}^{-}$ , Na^+^, K^+^, Cl^−^ and $SO_{4}^{2-}$ in ground water was investigated in Abhar rural area, Zanjan province, Iran.
• Continuing the ascending trend of the parameters concentration and declining the quality of water resources and incompatibility with Iranian drinking water standard can lead to significant health risks.
• Tracking the trend changes, investigating the reasons and preventive measures are important

## 1 Data

**Fig. 1:**
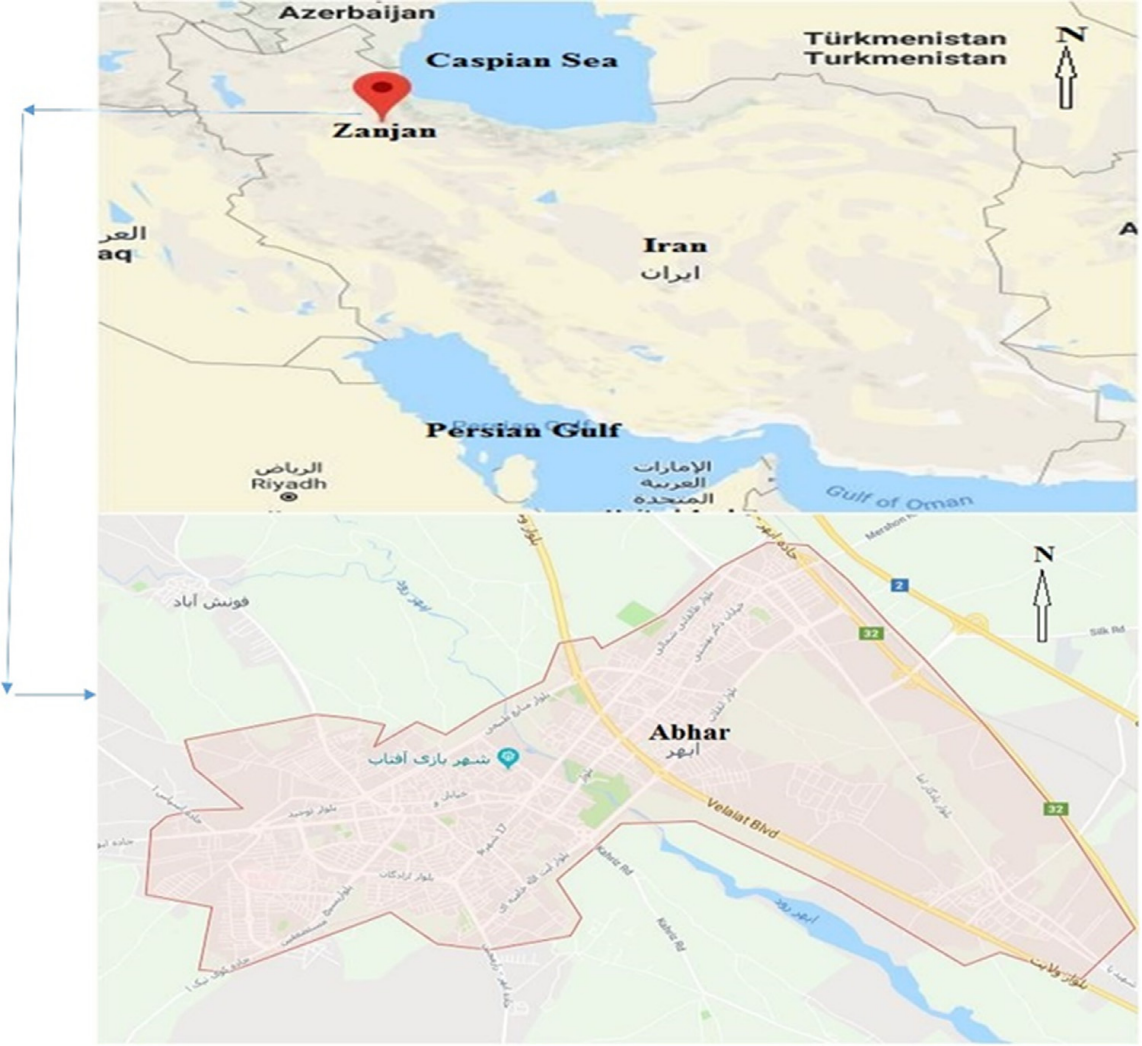
Location of the study area in Abhar city, Zanjan province, Iran.

**Chart 1:**
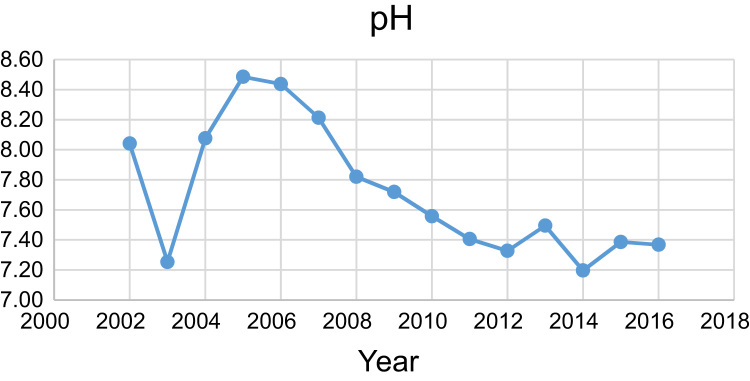
pH trend for groundwater in Abhar city.

**Chart 2:**
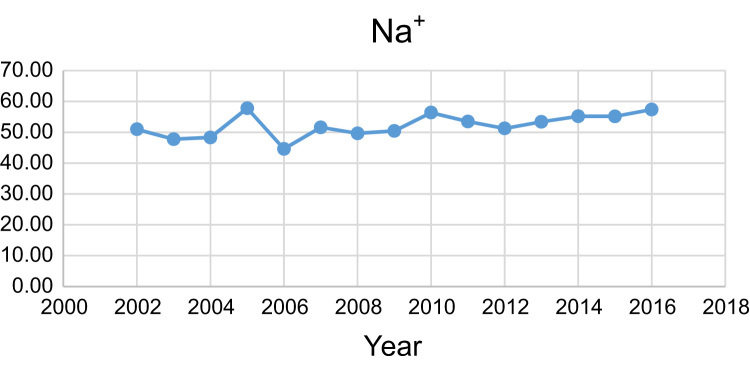
Na^+^ trend for groundwater in Abhar city.

**Chart 3:**
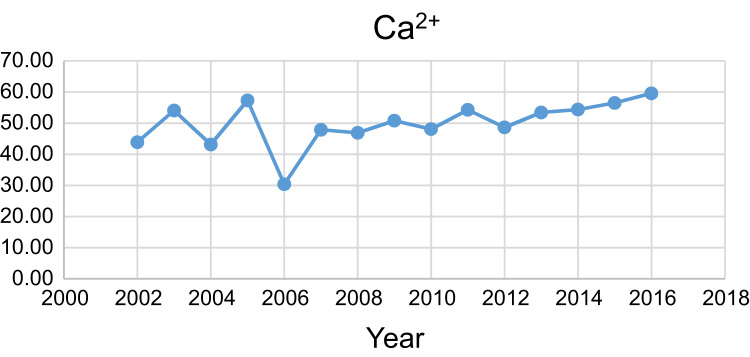
Ca^2+^ trend for groundwater in Abhar city.

**Chart 4:**
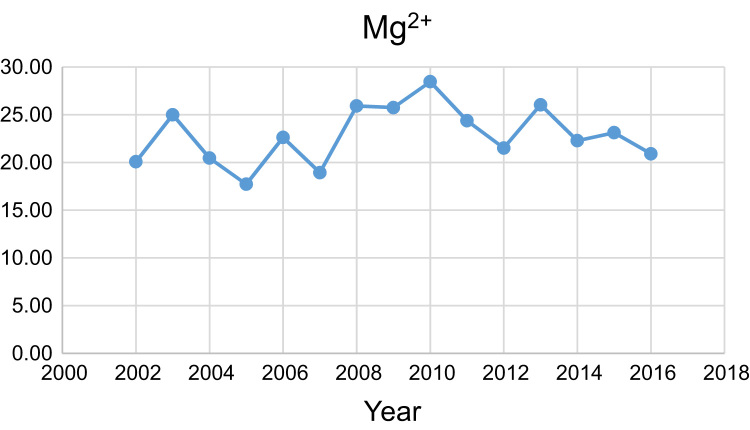
Mg^2+^ trend for groundwater in Abhar city.

**Chart 5:**
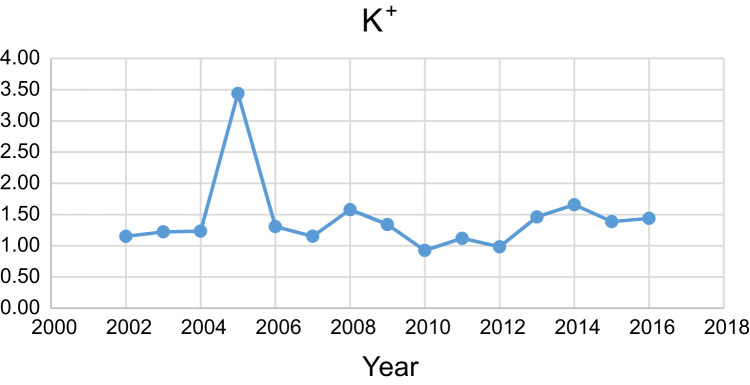
K^+^ trend for groundwater in Abhar city.

**Chart 6:**
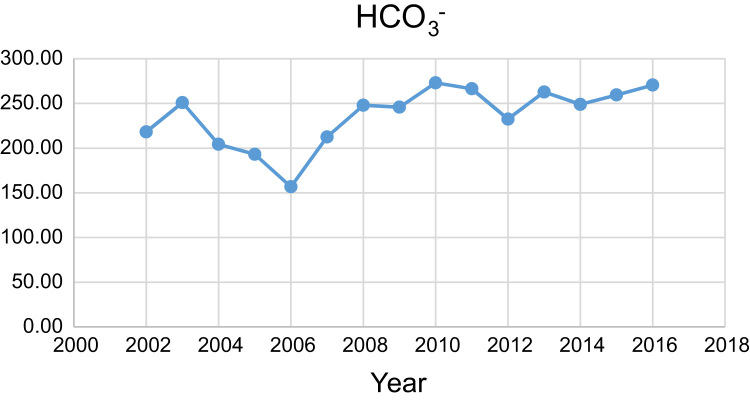
$HCO_{3}^{-}$ trend for groundwater in Abhar city.

**Chart 8:**
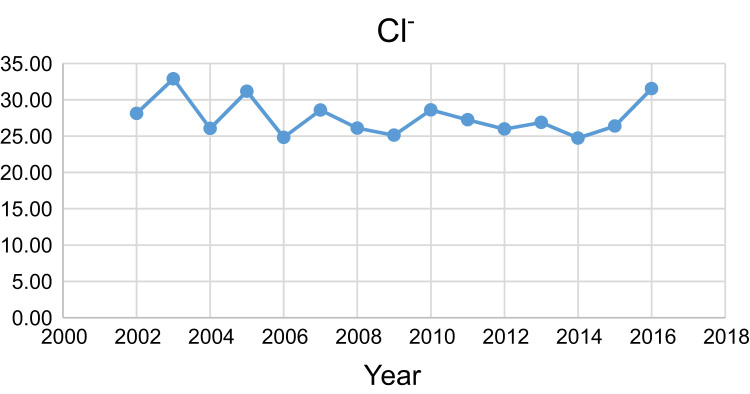
Cl^−^ trend for groundwater in Abhar city.

**Chart 9:**
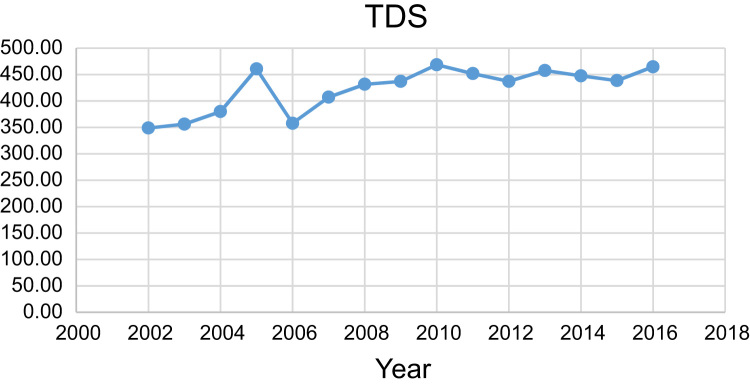
TDS trend for groundwater in Abhar city.

**Chart 10:**
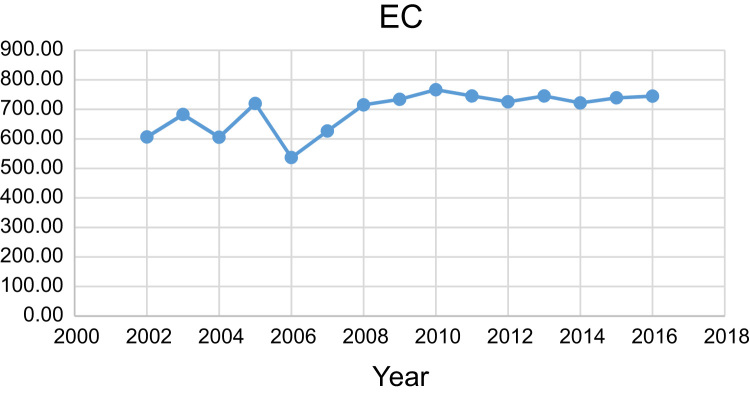
EC trend for groundwater in Abhar city.

Data presented here deal with monitoring of physical and chemical including pH, Na^+^, Ca^2+^, Mg^2+^, K^+^, EC, TDS, $HCO_{3}^{-}$, $SO_{4}^{2-}$, Cl^−^, and TH as in Abhar County, Zanjan Province, Iran. Fig. 1 shows the study area and the sampling points. A summary of Water quality characteristics and correlation of the parameters with fluoride are presented in Table 1, Table 2 respectively. Chart 1, Chart 2, Chart 3, Chart 4, Chart 5, Chart 6, Chartt 7, Chart 8, Chart 9, Chart 10 show trend in some parameter in the years (2002–2016)

**Chartt 7:**
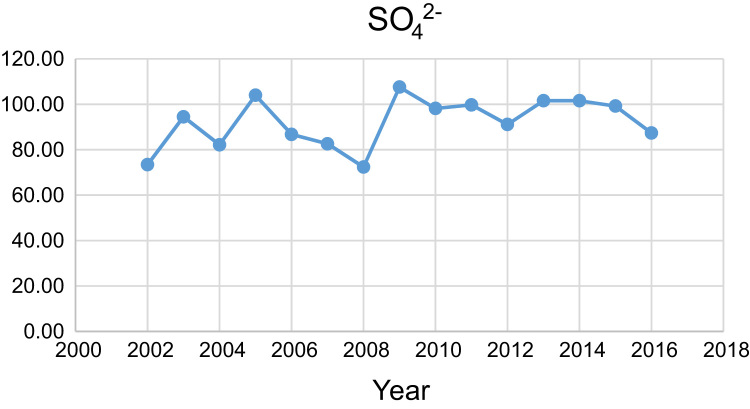
$SO_{4}^{2-}$ trend for groundwater in Abhar city.

**Table 1:** Chemical analysis report of water quality of drinking water resource of Abhar city.

**Year**	**pH**	**Na**^**+**^	**Ca**^**2+**^	**Mg**^**2+**^	**K**^**+**^	$\mathrm{HC}O_{3}^{-}$	SO_4_^2−^	**Cl**^**−**^	**TDS**	**EC**	**TH**
		**(mg/L)**	**(mg/L)**	**(mg/L)**	**(mg/L)**	**(mg/L)**	**(mg/L)**	**(mg/L)**	**(mg/L)**	**(μmhos/cm)**	**(mg/L as CaCO**_**3**_**)**
2002	8.04	50.97	43.90	20.07	1.15	218.19	73.40	28.13	348.79	606.21	192.69
2003	7.25	47.75	54.09	25.00	1.22	250.91	94.48	32.90	356.10	682.50	238.52
2004	8.08	48.31	43.15	20.45	1.23	204.24	82.16	26.05	379.93	605.52	192.40
2005	8.49	57.79	57.35	17.72	3.44	193.09	103.99	31.18	460.82	719.82	216.59
2006	8.44	44.61	30.39	22.62	1.31	156.75	86.76	24.83	357.70	536.68	169.45
2007	8.21	51.60	47.88	18.93	1.15	212.51	82.60	28.59	407.16	626.77	197.93
2008	7.82	49.65	46.90	25.91	1.58	247.96	72.37	26.10	431.63	715.19	224.34
2009	7.72	50.40	50.81	25.75	1.34	245.81	107.58	25.12	437.05	733.99	233.40
2010	7.56	56.38	48.09	28.46	0.92	273.10	98.17	28.61	468.52	766.44	237.83
2011	7.41	53.48	54.29	24.38	1.12	266.41	99.74	27.25	451.87	745.15	236.46
2012	7.33	51.24	48.64	21.51	0.98	232.45	91.12	25.97	437.20	725.52	226.67
2013	7.49	53.39	53.43	26.04	1.46	262.81	101.58	26.88	457.64	745.11	241.16
2014	7.20	55.18	54.38	22.28	1.66	248.97	101.57	24.74	447.59	721.77	228.02
2015	7.39	55.15	56.51	23.12	1.39	259.47	99.22	26.38	438.60	738.78	236.82
2016	7.37	57.36	59.58	20.91	1.44	270.51	87.36	31.54	464.71	744.55	235.25
Mean	7.70	51.83	49.53	22.97	1.33	236.76	92.27	27.09	422.55	691.57	219.95
Max	10.84	207.46	198.00	112.17	29.25	1073.60	420.00	159.75	1856.00	2420.00	880.00
Min	5.61	0.00	0.00	0.00	0.00	0.00	0.00	0.00	2.55	177.00	40.50
S.D	0.55	30.29	23.75	13.11	1.69	112.21	76.42	16.57	191.06	297.24	95.99
WHO Guide line	6.5–8.5	400.00	250.00	150.00	–	–	200.00	200.00	500.00	–	200.00
1053IR	6.5–8.5	200.00	300.00	30.00	–	–	250.00	250.00	1000.00	–	200.00

**Table 2:** Pearson Correlation of the chemical parameters.

	**pH**	**Ca**^**2+**^	**Mg**^**2+**^	**Na**^**+**^	**K**^**+**^	$\mathrm{HC}O_{3}^{-}$	$SO_{4}^{2-}$	**CL**^**−**^	**TDS**	**EC**	**TH**
**pH**	1										
**Ca**^**2+**^	− 0.547**	1									
**Mg**^**2+**^	− 0.247**	0.461**	1								
**Na**^**+**^	− 0.226**	0.519**	0.433**	1							
**K**^**+**^	− 0.069	0.186**	0.326**	0.179**	1						
$\mathrm{HC}O_{3}^{-}$	− 0.545**	0.73**	0.747**	0.424**	0.31**	1					
$SO_{4}^{2-}$	− 0.171**	0.487**	0.365**	0.804**	0.076*	0.116**	1				
**CL**^−^	− 0.218**	0.624**	0.574**	0.734**	0.258**	0.582**	0.536**	1			
**TDS**	− 0.322**	0.722**	0.681**	0.775**	0.242**	0.666**	0.665**	0.747**	1		
**EC**	− 0.443**	0.811**	0.758**	0.824**	0.28**	0.767**	0.701**	0.796**	0.911**	1	
**TH**	− 0.489**	0.865**	0.837**	0.55**	0.291**	0.86**	0.495**	0.697**	0.824**	0.922**	1

⁎⁎ Correlation is significant at the 0.01 level (2-tailed).

⁎ Correlation is significant at the 0.05 level (2-tailed).

## 2 Experimental design, materials and methods

### 2.1 Study area description

Abhar is the capital of Abhar city in Zanjan province in Iran. Abhar city is located in Zanjan province at UTM coordinates of *X* = 49.25–48.35 east longitude and *Y* = 36.45–35.50 north latitude. The climate of the study area is semiarid, and the precipitation is 300 mm per year. Also the air's highest and lowest temperatures are 38 °C and − 5.15 °C, respectively, with an annual average of 12.7 °C (Fig. 1).

### 2.2 Data collection

The required data were collected from the results recorded in the water in the Iran Water resources management Company during the years 2002–2016. In this study, 750 samples were analyzed by descriptive and analytical statistics (correlation coefficients) in 15 years (Table 3). The important major cations and anions in water samples were analyzed following a standard method (APHA 2008) [1], [2], [3], [4], [5], [6], [7], [8], [9], [10], [11], [12].

**Table 3:** Number of samples in years studied (2002–2016).

Year	Number of samples
2002	39
2003	30
2004	56
2005	11
2006	57
2007	61
2008	43
2009	78
2010	27
2011	54
2012	56
2013	55
2014	56
2015	56
2016	51
